# Case of Third Dentition

**Published:** 1845-09

**Authors:** 


*Case of Third Dentition.*—A mechanic, residing in Boston, who had the last of a bad set of teeth extracted within a few months, has since been surprised by the appearance of several new teeth, which are just protruding their sharp edges through the gums. The prospect, at present, is, that the third set will not be either numerous or symmetrical, but perhaps of considerable utility in his old age. There is no appearance of a grinder, the new teeth being nearly on the alveolar arch. The jaws cannot be brought in contact—nor have they been since the remnant of the second set was taken out. As the gentleman has promised to call, from time to time, to give us an opportunity of observing the progress of this anomalous dentition, we shall not fail to mention the appearance of the favored individual, when nature has completed the curious operation she has commenced, so much out of the ordinary course of her proceedings, in his case.—*Ibid.*

